# Electrophysiological correlates of the processing of different self-aspects of handwritten names

**DOI:** 10.1038/s41598-019-45849-x

**Published:** 2019-07-01

**Authors:** Reiko Sawada, Motomi Toichi, Nobuo Masataka

**Affiliations:** 1 Graduate School of Medicine, Kyoto University, 53 Shogoin Kawahara-cho, Sakyo-ku, Kyoto, 606-8507 Japan; 2 Organization for Promoting Developmental Disorder Research, 40 Shogoin Sanno-cho, Sakyo-ku, Kyoto, 606-8392 Japan; 3 Primate Research Institute, Kyoto University, 41-2 Kanrin, Inuyama, Aichi 484-8506 Japan

**Keywords:** Human behaviour, Perception

## Abstract

Humans recognize the self in various visual domains, such as faces, names, and motions, as well as in products, such as handwritten letters. Previous studies have indicated that these various domains of self are represented differently in the brain, i.e., domain-specific self-representation. However, it remains unclear whether these differences in brain activation are due to the processing of different visual features or to differential self-processing among the domains, because the studies used different types of visual stimuli. The present study evaluated event-related potentials (ERPs) while participants were presented with their own and others’ names generated by the participants themselves or someone else. Therefore, the visual stimuli included two domains of self-related information, name and motor agent, but only one type of stimulus (handwritten names). The ERP results show that the amplitudes of the P250 component (250–330 ms) in the posterior regions were smaller for self-generated handwritten names than for non-self-generated handwritten names. The results also show that the amplitudes of the P300 component (350–500 ms) were larger for the self-name than for the non-self-name. These results suggest domain-specific processing of self-related information regarding the name and agent of handwritten stimuli.

## Introduction

The self is recognized in various visual domains, such as the face and body parts, in self-related words including written names, in motion, and in products such as handwritten letters. Although several previous studies have reported that visual self-recognition is a higher-level social ability implemented by the general module for self-processing^1,2^, several studies have also suggested that there are differences among the domains of the self in the neural self-representations in the brain, i.e., specificity of visual self-representations. Comprehensive reviews have proposed a distinction between the physical and mental aspects of visual-self representation^3,4^. This has been demonstrated by previous studies showing domain specificity for visual self-representations, such that brain activity in the cortical midline structures is involved in self-processing and social aspects, including self-appraisal^5^ and the judgment of personal traits^6^, whereas fronto-parietal regions, particularly in the right hemisphere, are involved in bodily-self-recognition, including recognition of self-face^7,8^ and body parts^9^.

Several studies have directly compared the self-processing of multiple visual stimuli. For example, a neuroimaging study found differences in brain activation related to self-processing between face and name^10^. Electrophysiological studies have identified different temporal and/or spatial brain activities in response to different domains of self-information^11–13^. Although similarities among the domains have also been observed, information related to the self in different domains engaged attentional allocation^14^; indeed, it seems that previous findings support the existence of domain-specific visual self-representation.

However, because the abovementioned studies compared different types of visual stimuli in terms of self- and non-self-relevance (e.g., face vs. name), it remains unclear whether these differences are due to different types of visual processing (e.g., face processing vs. name processing) or to the differential processing of self-related information (e.g., face-specific self-processing vs. name-specific self-processing). Thus, the goal of the present study was to investigate the existence of domain-specific self-processing using stimuli that were based on a common visual input but comprised two types of self-related information. For this purpose, handwritten names were selected as stimuli because they include two aspects of self-related information: name (self-name) and motor agent (self-generated handwriting).

A name is a symbolic item that represents the self. Event-related potential (ERP) studies have demonstrated that, compared to non-self-names, self-names augment the P300 amplitude, which is a positive deflection that occurs mainly in midline brain regions at approximately 350–500 ms after stimulus onset^12,15–17^. Because it has been suggested that the amplitude of the P300 component reflects the extent to which participants allocate attentional resources to a stimulus^18^, previous findings of enhanced P300 amplitudes have been interpreted to mean that written self-names effectively capture visual attention due to self-relevance.

Self-handwritten characters also represent the self, but this is essentially independent of one’s own name. Recent electrophysiological studies have shown that self- and non-self-generated handwritten characters elicit different patterns of brain activation in the right posterior regions at approximately 300 ms after stimulus onset^19,20^. Additionally, Sawada *et al*.^20^ reported that the amplitude of P250, a positive component occurring in the posterior regions at around 250–350 ms after stimulus onset, was smaller for self-generated handwritten characters than for non-self-generated handwritten characters. Some studies have demonstrated that P250 amplitudes reflect the degree of the perceptual load in accessing the stored representations that are developed through long-term experiences with stimuli^21,22^. Therefore, smaller P250 amplitudes for self- versus non-self-handwritten stimuli may indicate that self-generated handwritten characters demand less perceptual load than do non-self-generated handwritten characters in terms of retrieving and accessing the stored structural representations.

This study used ERPs to investigate the neural correlates underlying the name- and agent-specific self-processing of static handwritten names. ERP measurements are a powerful tool for assessing the temporal features of stimulus processing due to their sensitive time resolution. This approach also allows investigation of the information processing of the presented stimuli prior to the occurrence of behavioral responses. If the self-relevance of name and agent affects ERP components in an identical manner, this would support the notion of a domain-general visual representation and a general module for self-processing. If the ERP components were differentially affected, this would support the notion of domain-specific visual self-representations for name and agent. Based on the abovementioned studies on self-processing of names^12,15–17^ and handwritten characters^20^, we would expect positive ERP results. If there were name-specific and handwriting-agent-specific self-representations, we would expect a self-name to enhance the P300 component compared to a non-self-name. We would also expect that P250 amplitudes in the right posterior regions would be smaller for self-generated handwritten stimuli than for handwritten stimuli generated by another individual.

Additionally, the P100 component, a positive component occurring in the occipito-parietal region at around 100–150 ms after stimulus onset, was analyzed to assess the effects of processing the basic visual features of stimuli, including size and luminance^23^. The pN1 and pP1 components, which are negative and positive components, respectively, occurring in the prefrontal regions from 80 to 230 ms, were also analyzed to assess the effects of perceptual and sensorimotor awareness^24^. These components are known to reflect the top-down perceptual processing of any stimulus^24,25^. We expected that the self-related processing of handwritten names would not be associated with these basic visual features. Moreover, we analyzed the N170 component, a negative component occurring in the occipito-parietal region at around 170 ms after stimulus onset, that reflects structural encoding processing^26^. Some previous studies showed that self-related stimuli enhanced the N170 component (e.g., self-face^27^), whereas other studies found no differences in the N170 amplitudes in response to self- and non-self-related stimuli^20,22^; therefore, the sensitivity of the N170 component to self-processing remains ambiguous.

## Results

### Behavioral data

The mean accuracy rate and RTs under the name and agent task conditions are summarized in Table 1.

**Table 1:** Mean (±SE) accuracy (%) and reaction times (ms) for self-, close-associate, notable, and unknown names generated by the participant him/herself (self) and someone else (non-self) in the name and agent tasks.

	Self				Non-self			
	Self	Close	Notable	Unknown	Self	Close	Notable	Unknown
**Accuracy**								
Name task	98.9 (0.5)	94.7 (1.0)	95.9 (1.4)	97.8 (0.7)	97.8 (0.7)	94.1 (1.4)	96.7 (1.4)	98.7 (0.5)
Agent task	98.4 (0.6)	94.4 (1.3)	90.0 (2.9)	93.3 (2.0)	92.2 (2.8)	92.7 (2.3)	97.1 (0.9)	95.1 (1.5)
**Reaction time**								
Name task	538.3 (25.6)	579.9 (30.2)	594.7 (28.4)	590.8 (23.0)	547.3 (32.5)	588.3 (25.9)	587.8 (22.4)	597.0 (24.5)
Agent task	787.7 (76.3)	855.8 (78.6)	864.5 (73.9)	872.6 (87.0)	752.6 (47.8)	853.2 (82.0)	761.2 (53.2)	776.0 (46.4)

#### Accuracy

*T*-tests revealed that the participants correctly recognized the self-relevant information about both name and agent under all stimulus conditions at a rate above chance (50%) in both the name (*t*(22) > 32.5, *p* < 0.001) and agent (*t*(22) > 13.7, *p* < 0.001) tasks. A three-way analysis of variance (ANOVA) with name, agent, and task as factors revealed significant main effects of task (*F*(1, 22) = 7.3, *p* < 0.05) and name (*F*(3, 66) = 3.9, *p* < 0.05) and a significant interaction between name and agent (*F*(3, 66) = 6.0, *p* < 0.005). The other main effects and interactions were not statistically significant (*F*s < 2.6, *p*s > 0.06).

The significant main effect of task indicated that participants responded more accurately in the name task than in the agent task. A follow-up analysis of the interaction between name and agent revealed a significant simple-effect of name for self-generated, but not non-self-generated, handwritten stimuli. Multiple comparisons with Bonferroni correction indicated that self-name stimuli were responded to more accurately than were close, notable, and unknown names under the self-generated handwritten condition (*t*(132) > 3.1, *p* < 0.05). However, no difference was found among others’ names (*t*(132) < 2.2, *p* > 0.2).

#### RTs

A three-way ANOVA with name, agent and task as factors revealed significant main effects of task (*F*(1, 22) = 16.2, *p* = 0.001) and name (*F*(3, 66) = 6.89, *p* < 0.001). The other main effects and interactions were not statistically significant (*F*s < 1.9, *p*s > 0.1). These results indicate that the participants responded faster in the name task than in the agent task. Multiple comparisons for a main effect of name indicated that self-name was responded to faster than were close, notable, and unknown names (*t*(132) > 3.3, *p* < 0.05). No significant differences were found among non-self-names (*t*(132) < 1.3, *p* > 0.9).

### ERP data

#### *P250 (250–330* *ms) in the lateral posterior regions*

**Figure 1:**
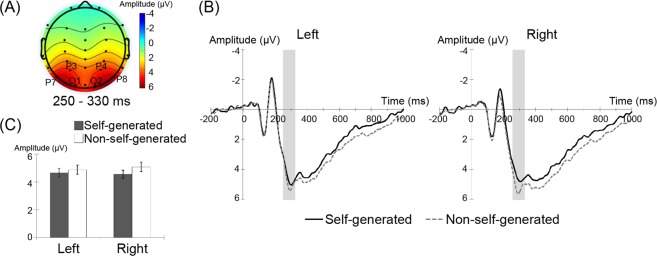
(**A**) Topographical distributions of the P250 component (250–330 ms) averaged across all experimental conditions. (**B**) Grand-average waveforms for self- (black solid lines) and non-self-generated (gray dashed lines) handwritten stimuli at the left (P7, P3, and O1) and right (P8, P4, and O2) posterior electrode sites. (**C**) Mean (±*SE*) P250 amplitudes in left and right posterior regions for self- and non-self-generated handwritten names.

The grand-average waveforms in the left (P7, P3, and O1) and right (P8, P4, and O2) posterior regions in response to self- and non-self-generated stimuli under each name condition and the mean amplitudes (±standard error (*SE*)) of the P250 component in the left and right posterior electrode sites in response to self- and non-self-generated handwritten names are shown in Fig. 1. A three-way ANOVA with name, agent, and site as factors revealed significant main effects of agent (*F*(1, 22) = 5.0, *p* < 0.05) and significant interactions between agent and site (*F*(2, 44) = 5.3, *p* < 0.05). No other main effects or interactions were statistically significant (*p* > 0.2). Follow-up analyses of the interaction between agent and site revealed a simple-effect of agent in the right electrode site (*F*(1, 44) = 9.0, *p* < 0.01) but not in the left hemisphere (*F*(1, 44) = 1.4, *p* > 0.2). The analyses showed that simple-effects of site for self- and non-self-generated handwritten names were not significant (*F*(1, 44) < 0.5, *p* > 0.5). Thus, the present results indicate that self-generated handwritten names elicited smaller P250 amplitudes than non-self-generated handwritten names in the right posterior regions.

#### *P300 (350–500* *ms) in the centro-parietal regions*

**Figure 2:**
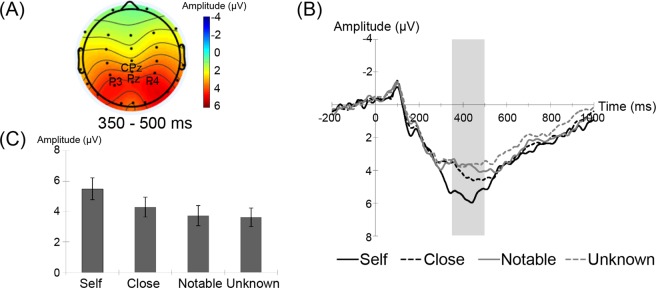
(**A**) Topographical maps of P300 (350–550 ms) averaged across all experimental conditions. (**B**) Grand-average waveforms for self- (black solid lines), close (black dashed lines), notable (gray solid lines), and unknown (gray dashed lines) name stimuli at the centro-parietal electrode sites (CPz, Pz, P3, and P4). (**C**) Mean (±*SE*) P300 amplitudes under the self-, close, notable, and unknown name conditions.

The grand-average waveforms in the centro-parietal regions and the mean amplitudes (±*SE*) of the P300 component in the regions are shown in Fig. 2 according to the name condition. A two-way ANOVA with name and agent as factors revealed a significant main effect of name (*F*(3, 66) = 10.3, *p* < 0.001). No other significant effects were observed (*p* > 0.09). Multiple comparisons with Bonferroni corrections for the main-effect of name revealed that the P300 amplitudes for self-names were larger than those for close (*t*(66) = 3.2, *p* < 0.05), notable (*t*(66) = 4.2, *p* < 0.005), and unknown (*t*(66) = 6.5, *p* < 0.001) persons’ names, but there was no significant difference among non-self-names (*t*(66) < 2.2, *p* > 0.2).

#### *P100 (100–150* *ms) and N170 (150–200* *ms) in the lateral posterior regions*

The grand-average waveforms and the mean amplitudes (±*SE*) of the P100 and N170 components in the left (TP7, P7, and O1) and right (TP8, P8, and O2) electrode clusters are shown in Fig. S1. A three-way ANOVA with name, agent, and site as factors revealed no main effects or interactions for the P100 amplitudes (*p* > 0.1) and N170 amplitudes (*p* > 0.1).

#### *pN1 (80–140* *ms) and pP1(160–230* *ms) in the frontal regions*

The grand-average waveforms, topographical maps, and mean amplitudes (±*SE*) of the pN1 and pP1 components in the frontal region (F3, F3, Fz, and FCz) electrode clusters are shown in Fig. S2. A two-way ANOVA with name and agent as factors revealed no main effects or interactions for the pN1 (*p* > 0.1) and pP1 (*p* > 0.05) amplitudes.

## Discussion

This study measured ERPs while participants viewed their own and others’ names in self-generated and non-self-generated handwritten forms to determine whether there was specificity in the processing of different aspects of self-relevance.

The ERP results show that non-self-generated handwritten names increased P250 (250–330 ms after stimulus onset) amplitudes in the posterior regions relative to self-generated handwritten names regardless of who the names represented. This result is consistent with previous findings showing differences between self-generated and non-self-generated handwritten characters in the posterior regions at around 300 ms after stimulus onset^19,20^. Because participants in the present experiment merely viewed their own handwritten names or names generated by another individual, the P250 amplitudes in the posterior regions may have been modulated by agent information without any explicit judgment of self-relevance. Thus, this result suggests that the ERP components occurring in posterior regions at approximately 300 ms after stimulus onset reflect the authorship of general handwritten forms. Given that P250 amplitudes reflect the activation of stored representations^21^ developed based on long-term experience^28^, self-generated handwritten forms, including self-handwritten names, require a lower perceptual load when matching visual inputs with stored representations of self-written products.

The ERP results also indicate that self-names enhanced P300 amplitudes (350–500 ms after stimulus onset) relative to close, notable, and unknown persons’ names under both self-generated and non-self-generated handwritten name conditions. This is consistent with the findings of previous studies that employed printed self- and non-self-names^12,15–17^. The present findings also show that P300 amplitudes increased not only when the names were presented in printed form but also when presented in handwritten form. Because the P300 amplitude is thought to reflect heightened attention^18,29^, our results indicate that written self-names more effectively captured visual attention than others’ names in written form. This finding is also in agreement with those of previous studies showing visual attentional capture of self-name using other experimental paradigms. For example, a greater reduction has been reported in the attentional blink^30^ and repetition blindness during rapid serial visual presentations^31^ in response to one’s own name compared with others’ names, suggesting enhanced visual attention to self-names compared to others’ names. Although some studies have argued that one’s own name does not effectively capture visual attention when the task is irrelevant to the self^32^, our results indicate that self-names capture visual attention even during the passive viewing of written names.

Most importantly, our results indicate that there were different visual representations of name and agent in terms of the handwritten name stimuli. Although this finding is consistent with previous studies that demonstrated domain-specific self-representations^10,11^, it remains unclear whether the previous results reflect differences in self-relevant information processing or differences in visual processing related to stimuli; this is because previous studies used different types of visual stimuli. To the best of our knowledge, this study is the first to investigate different aspects of visual self-representation using common stimuli with multiple dimensions of self-related information.

The present results are also consistent with accumulated evidence showing that visual self-representations correspond to physical as well as mental aspects of self ^4^. For example, fronto-parietal regions, especially in the right hemisphere, are involved in recognition of the bodily self, such as one’s own face^7,8^ and body parts^9^. Moreover, behavioral and neuroimaging studies have found that the products of self-generated motor actions, such as one’s own voice, are associated with right-lateralized brain activation^7,33,34^. Similarly, handwritten stimuli are generated by prior writing actions. It has been suggested that agent recognition of dynamic handwriting is linked to self-motor experience^35^. Previous studies have shown that variables related to an individual’s writing style, such as stroke order, direction, and pressure, influence subsequent recognition of static handwritten characters^36^. Moreover, previous ERP studies^20^ using handwritten characters revealed differences in P250 amplitudes in the right posterior regions and suggested that these results were due to individual differences in the kinematic information provided during writing. Taken together, the present and previous results suggest that self-related kinematic characteristics are embedded in handwritten stimuli and that information regarding one’s own handwriting might involve bodily self-processing.

Moreover, previous neuroimaging studies have shown that brain activations in the cortical midline structures might be involved in mental aspects of self recognition, such as self-appraisal^5^ and judgments of the self-relevance of words^6^. This region has also been reported to be associated with recognizing social relationships with others^37,38^. Our results showing an enhancement of P300 amplitudes while observing self-names versus others’ names may indicate that the processing of one’s own name is associated with mental self-representations or the processing of social significance. However, there were no differences in P300 amplitudes among the close, notable, and unknown persons’ names in the present study. In contrast, some studies have observed differences among others’ names in terms of self-relevance^12,16^, and some have reported no differences in brain activity in the centro-parietal sites at 300–750 ms in response to self-names and names of close associates^39^. Methodological differences may explain these inconsistent findings. First, our participants passively observed the handwritten name stimuli, and the tasks were irrelevant to the name stimuli, whereas participants in previous studies were asked to judge whether the names were familiar or unfamiliar or to engage in word-relevant tasks. Second, the names of family members or friends were used as stimuli for a close associate. Although the names of close associates were truly familiar to the participants in our study, there may have been variations among participants in the degree of closeness to the self.

There were no differences in N170 amplitudes in the posterior regions between self- and non-self-relevant stimuli in the present study. Several previous studies observed differences in N170 amplitudes between self- and non-self-relevant stimuli, such as self-versus non-self-faces^27^, whereas others reported no such differences^20–22^. The present results are consistent with the latter findings and support the view that the processing of self-relevance does not reflect the structural encoding stage^26^.

There were also no differences in the P100 component in the posterior regions among the stimulus conditions. P100 amplitudes are sensitive to the basic perceptual aspects of stimuli, such as size, brightness, and contrast^23^. Moreover, no significant difference was observed in the pN1 and pP1 amplitudes across stimulus conditions. These components were known to reflect top-down perceptual processing of stimuli^24,25^. In the present study, there were no differences in the means and standard deviations (*SD*s) of the luminance among the stimulus conditions, and the size of the writing was controlled; therefore, our results indicate that the processing of information related to the self-relevance of the name and agent of static handwritten names was not associated with the processing of basic perceptual factors or perceptual awareness.

The behavioral results indicate that participants responded more accurately and rapidly to their own names than to close, notable, and unknown person’s names, which is consistent with the results of the P300 amplitude analyses. Taken together, these findings suggest that heightened visual attention to self-name may facilitate recognition of one’s own name in terms of both accuracy and speed. However, there were several discrepancies between the behavioral and ERP results. It seems counterintuitive that there was no interaction between name and agent in the ERP results, even though there was a significant interaction with accuracy. Moreover, the RT results show that judgments of the self-relevance of agents required more time than did judgments of names, whereas the ERP results show no such differences in the time window that reflects the self-processing of the names and agents associated with handwritten stimuli. One possible explanation for these discrepancies is task differences. In the behavioral experiments, the participants were asked to judge the handwritten stimuli according to self-relevance, whereas they passively viewed the stimuli in the ERP experiment. Additional ERP recordings while making explicit self/other judgments would explain these discrepancies.

Several limitations of this study should be acknowledged. First, similarities in the shapes of the self-generated and non-self-generated handwritten name stimuli were not controlled in the present study. We found no differences in the basic visual features, such as the means and *SD*s of the luminance and visual complexity of self-generated and non-self-generated stimuli. A previous study reported that the P250 amplitudes in the right posterior regions were smaller in response to self-generated, compared with non-self-generated, handwritten characters even when the shapes were strictly stylized^20^. However, additional research with participant pairs with various degrees of similarity in their handwritten shapes will clarify the effect of shape similarity on the processing of self-generated handwritten forms. Second, participants passively viewed the handwritten names in the ERP experiment. An ERP study including a task relevant to the stimuli, such as one concerning the familiarity and self-relevance of a name or writer, would provide further information about the self-related processing of handwritten names. A previous study showed that a task in which participants judged whether the presented stimulus was their own handwritten character enhanced the ERP component occurring in the anterior regions at 350–500 ms after stimulus onset^20^. Some recent studies also showed that stimulus-related tasks modulated the pP2 amplitudes; pP2 is a positive component that occurs in the prefrontal regions at around 400 ms, which are involved in decision making^24,25^. Thus, a future ERP study using stimulus-related tasks would provide further evidence of the neural correlates underlying recognition of different aspects of self.

In summary, we measured ERPs while participants viewed their own and others’ names, which were written either by the participants themselves or by another individual. The handwritten stimuli included two domains of self-related information, name and motor agent, but the visual input was physically controlled using one type of stimulus, handwritten names. The ERP results show differences in brain activation between self-generated and non-self-generated handwritten forms at 250–330 ms after stimulus onset in the posterior regions. Moreover, there were differences in brain activation between self-names and non-self-names at 350–500 ms after stimulus onset. These results support the notion of domain-specific processing of self-related information.

## Materials and Methods

### Participants

In total, 24 Japanese individuals (11 males and 13 females; *M* ± *SD* age, 27.3 ± 5.3 years) participated in the ERP and behavioral experiments. They were paid for their participation. The sample size of this study was determined by an a priori power analysis using G∗Power (ver. 3.1.9.4)^40,41^ based on eight measurements, an *α* of 0.05, a power (1-*β*) of 0.95, an *ε* of 0.143, and medium-sized effects *f* of 0.25^40^. The result indicated that more than 23 respondents were necessary. All participants were right-handed, as assessed by a handedness inventory^42^, and had normal or corrected-to-normal visual acuity. Data from one participant were excluded from the analyses because this individual could not identify self-handwritten stimuli in the behavioral experiment.

### Stimuli

All stimuli were Japanese names presented in Chinese characters and/or Japanese syllables. Ten patterns of names written by either the participant (self-generated) or someone else (non-self-generated) under four conditions (name of self, names of persons close to the participant (selected from friends or family members), names of notable people (selected from famous persons, e.g., politicians, athletes), and names of hypothetical persons (conventional names created by an author)) were prepared for the ERP and behavioral experiments. Written names generated by different participants, none of whom participated in the ERP or behavioral experiments, were used as non-self-generated names.

#### Writing session

The writing sessions were carried out more than 2 weeks prior to the ERP and behavioral experiments. First, participants were asked to provide the experimenter with the names of several (at least two) friends and family members. Then, names of two persons with first and last names that differed from those of the participant were selected for writing as the close-person stimuli. Participants were also presented with a list of names written in Chinese characters and Japanese syllable characters, which were shown in Mincho typeface. These included 16 names of actual notable people (e.g., Japanese politicians and athletes) and 16 conventional but hypothetical Japanese names. Participants were asked to rate their level of familiarity with these individuals on the following scale: (1) I do not know the person at all; (2) I have seen or heard the name but cannot identify the person; (3) I can identify the person by name but I have never met him/her; and (4) I am acquainted with the person. The names of two notable and two unknown people were selected according to their responses (ratings of 3 and 1, respectively). Ultimately, seven names were selected to be written: the participant’s own name and two names each from the close, notable, and unknown categories. All first and last names differed from one another.

**Figure 3:**
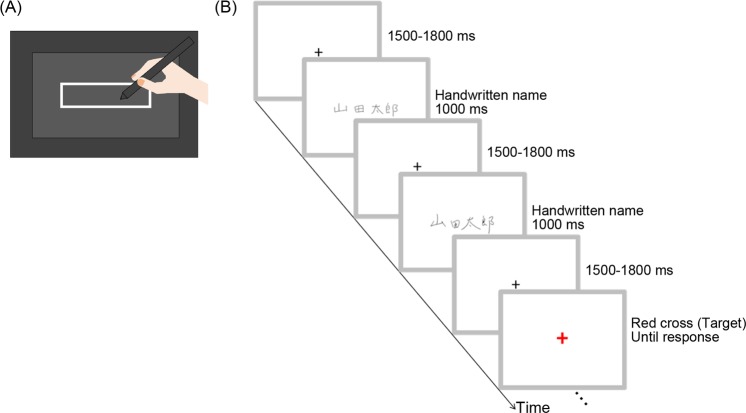
Schematic images of the writing apparatus (**A**) and stimulus presentation in the event-related potential experiment (**B**).

Then, participants were asked to write each name on a 9.5-cm wide × 3-cm high square grid using a graphics tablet (Intuos4 PTK-640, Wacom) implemented on a Windows computer (Precision M4800, Dell). The monitor was not visible to participants to prevent them from observing the stimuli prior to the ERP experiments. Figure 3A presents an image of the apparatus from the participants’ perspective. Each participant wrote out the names until 10 patterns were successfully obtained for each name. Participants were asked to write the name according to the experimenter’s verbal instructions, but they could see the list and confirm the characters. The order of the name stimuli was randomized.

After the self-generated handwritten stimuli were completed, we asked the participants who did not participate in the ERP/behavioral experiment to write the names to be used as non-self-generated stimuli. The names were written in the same manner as those written by the participants in the experiment. The pairs of participants who wrote self- and non-self-generated stimuli were determined pseudo-randomly so that the gender of these participants was counterbalanced.

#### Stimulus preparation

Each name was centered in the square using Photoshop CS6 (Adobe). The visual angle subtended by each stimulus was approximately 8.5° in width and 2.6° in height. Neither the self-name and three non-self-name (close, notable, and unknown persons) stimuli nor the self-generated and non-self-generated names differed from one another in basic visual features. We confirmed that there were no significant differences in mean (*p* > 0.09) and *SD* (*p* > 0.15) of the luminance across stimulus conditions using a two-way analysis of variance (ANOVA) with name (self, close, notable, and unknown) and agent (self and non-self) as factors. Moreover, to assess differences in visual complexity among the stimulus conditions, we analyzed perceptual visual complexity using graphics interchange format (GIF) compression, which has been established as a predictor of perceptual visual complexity^43^. A two-way ANOVA with name and agent as factors revealed no effects or interactions (*p* > 0.13), indicating no differences in the visual complexity of stimuli.

### Experimental procedure

At least 2 weeks (mean ± *SD*: 27.3 ± 12.4 days) after the writing session, the participants completed the ERP and behavioral experiments.

#### ERP experiment

Each participant was seated 65 cm from an 18.1-inch display monitor (AS4612UT, Iiyama) in a dimly lit room. Stimulus presentation was controlled using the Presentation software (Neurobehavioral System) implemented on a Windows computer (HP xw4300 Workstation, Hewlett-Packard Company).

The ERP experiment comprised eight blocks of 88 trials, with a short break between each block; 40 trials involved names generated by the participant, 40 trials involved names generated by an unknown person, and 8 trials involved a red cross. All name conditions had the same number of trials (i.e., 20 trials; 10 patterns of self-, close, notable, and unknown names under two agent conditions, i.e., self- and non-self-generated conditions). The order of stimulus presentation was randomized within each block.

The stimulus presentation procedure is shown in Fig. 3B. Each trial began with a fixation cross (0.6° × 0.6°) presented at the center of the screen for 1,300*–*1,700 ms. Next, a handwritten name was presented for 1,000 ms or a red cross (1.3° × 1.3°) was presented until the participant responded. All participants were asked to maintain their gaze on the fixation cross, carefully look at the stimuli, and respond to the red cross by pressing the right control button of a keyboard (KU-0316, Hewlett-Packard Company) as quickly and accurately as possible. Thus, participants were asked to passively view the name stimuli; the task was constructed to maintain the visual attention of participants. The mean ± *SD* reaction time (RT) corresponding to target detection was 782.6 ± 501.6 ms. No feedback was provided to participants regarding the accuracy of their responses.

#### Behavioral experiment

After the ERP experiment, each participant engaged in two behavioral tasks that assessed self-relevance in terms of person name (name task) and the author of the handwritten name (agent task). As in the ERP experiment, participants were seated 65 cm from the display monitor in a dimly lit room, and the presentation of stimuli was controlled using the same equipment.

Each task comprised two blocks of 80 trials, with a short break between blocks; 40 trials involved self-names and non-self-names (close, notable, and unknown persons) generated by the participant, and 40 trials involved names generated by an unknown person. The order of stimulus presentation was randomized within each block. Each trial began with a fixation cross (0.6° × 0.6°) presented at the center of the screen for 1,300–1,700 ms. Next, a handwritten name was presented until the participant responded. All participants were instructed to maintain their gaze on the fixation cross, carefully look at each stimulus, and indicate whether the person referenced by the name presented was an acquaintance or non-acquaintance (name task) or whether the name presented was written by the participant (agent task) by pressing the left or right control buttons of a keyboard (KU-0316, Hewlett-Packard Company) as quickly and accurately as possible. No feedback was provided to participants regarding the accuracy of their responses. The order of tasks and button positions was counterbalanced among the participants.

### Electroencephalography recording

Continuous electroencephalography (EEG) data were recorded from 30 scalp sites (Fp1, Fp2, F7, F8, F3, F4, Fz, FT7, FT8, FC3, FC4, FCz, T7, T8, C3, C4, Cz, TP7, TP8, CP3, CP4, CPz, P7, P8, P3, P4, Pz, O1, O2, and Oz) based on the International 10–20 system, using Ag-AgCl active electrodes (EasyCap) mounted on an elasticated cap. Electrooculogram (EOG) activity was monitored by electrodes placed on the bilateral canthi and above and below the left eye. A reference electrode was placed on the nose, and an electrode on the middle of the forehead served as the ground. Electrode impedance was kept below 5 kΩ, and the sampling rate was set at 1 kHz using the NeuroScan NuAmps System (Compumedics NeuroScan). All data were stored on a hard disk.

### Data analyses

All statistical tests were performed using IBM SPSS 22 software (IBM), and *p* values < 0.05 were considered to indicate statistical significance.

#### Behavioral data

The mean accuracy and RT for correct responses were calculated for each condition. Trials under the name familiarity and handwriting agency conditions that were ± 3 *SD*s from the mean RT were excluded from the analyses (mean ± *SD*: 1.9 ± 0.9 trials under the name condition; 3.1 ± 1.7 trials under the agent condition). The data were then subjected to a two-way repeated-measures ANOVA with name (self, close, notable, and unknown), agent (self and non-self), and task (name and agent) as the within-subject factors for each task condition. Simple-effects tests were performed to examine significant interactions. When higher-order interactions were significant, the main effects or lower-order interactions were not interpreted^44^. Multiple comparisons with Bonferroni corrections were performed to identify significant effects of name.

#### ERP data

ERPs were calculated with EEGLAB 13.1.1b^45^ (http://www.sccn.ucsd.edu/eeglab) implemented using Matlab R2012a (MathWorks). All EEG data were filtered offline with a band-pass of 0.1–30 Hz and segmented to obtain epochs starting at 200 ms prior to stimulus onset and ending at 1,000 ms after stimulus onset. Pre-stimulus data (from −200 to 0 ms) gathered while the fixation point was presented served as baseline data. Grand-average waveforms were calculated after trials with artifacts (>75 μV) were automatically rejected (*M* ± *SD* = 10.6 ± 8.1 trials, 13.2 ± 9.9%); the numbers of artifact-contaminated trials did not differ across conditions (*p* > 0.09) according to a two-way ANOVA with name (self, close, notable, and unknown) and agent (self and non-self) as factors.

The electrodes were selected for analyses based on maps of the topographical distribution of recorded brain activity in the time windows corresponding to given ERP components averaged across all experimental conditions^46^. The mean amplitudes of P250 (250–330 ms) components at the lateral posterior electrode sites (left: P3, P7, and O1; right: P4, P8, and O2) were subjected to a three-way ANOVA with name (self, close, notable, and unknown), agent (self and non-self), and site (left and right) as within-subject factors. The mean amplitudes of the P300 component (350–500 ms) at the centro-parietal electrode sites (CPz, Pz, P3 and P4) were subjected to a two-way ANOVA with name (self, close, notable, and unknown) and agent (self and non-self) as within-subject factors.

Additionally, the mean amplitudes of the P100 (100–150 ms) and N170 (150–200 ms) components at the lateral occipito-parietal electrodes (left: TP7, P7, and O1; right: TP8, P8, and O2) were subjected to a three-way ANOVA with name (self, close, notable, and unknown), agent (self and non-self), and site (left and right) as within-subject factors. Moreover, the mean amplitudes of the pN1 (80–140 ms) and pP1 (160–230 ms) components at the frontal electrodes (F3, Fz, F4, and FCz) were subjected to a two-way ANOVA with name (self, close, notable, and unknown) and agent (self and non-self) as within-subject factors.

A simple-effects test was performed to examine significant interactions. When higher-order interactions were significant, main effects or lower-order interactions were not interpreted^44^. The effects of name and electrode were analyzed using multiple comparisons with Bonferroni corrections.

### Ethics statement

All participants provided written informed consent. All experimental procedures were approved by the Ethics Committee of the Primate Research Institute of Kyoto University (numbers 2016-06 and 2017-02), and all experiments were performed in accordance with the Declaration of Helsinki.

## Supplementary information

Figure S1 and S2

## Data Availability

The datasets generated and/or analyzed during the current study are available from the corresponding author.
