# Supplementary material for: Electrophysiological correlates of the processing of different self-aspects of handwritten names
Source: Sci Rep. 2019 Jul 1;9:9432. doi: 10.1038/s41598-019-45849-x (PMC6602963; doi:10.1038/s41598-019-45849-x)
Supplement: Supplementary file 1 — Figure S1 and S2 [file 41598_2019_45849_MOESM1_ESM.docx]

**Electrophysiological correlates of the processing of different self-aspects of handwritten names**

Reiko Sawada^1,2*^, Motomi Toichi^1,2^, and Nobuo Masataka^3^

^1^Graduate School of Medicine, Kyoto University, Yoshida-Konoe-cho, Sakyo-ku, Kyoto, Japan 606-8501.

^2^Organization for Promoting Developmental Disorder Research, 40 Shogoin-Sanno-cho, Sakyo-ku, Kyoto, Japan 606-8392.

^3^Primate Research Institute, Kyoto University, 41-2 Kanrin, Inuyama, Aichi, Japan 484-8506.

*Corresponding author: sawada.reiko.3z@kyoto-u.ac.jp

**
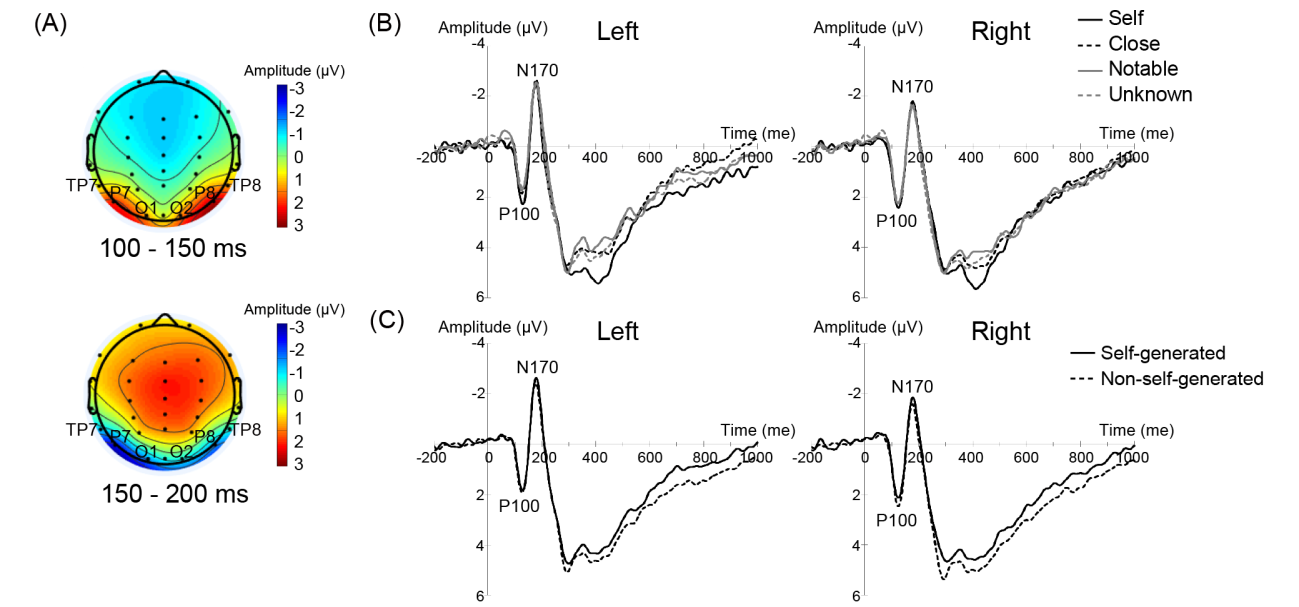
**

**Figure S1. P100 and N170 components.** (A) Topographical maps of P100 (100–150 ms) and N170 (150–200 ms) averaged across all experimental conditions. Grand-average waveforms for self- (black solid lines), close (black dashed lines), notable (gray solid lines), and unknown (gray dashed lines) name stimuli (B) and for self-generated (black solid lines) and non-self-generated (black dashed lines) stimuli (C) at the left (TP7, P7, and O1) and right (TP8, P8, and O1) posterior electrode sites.

**
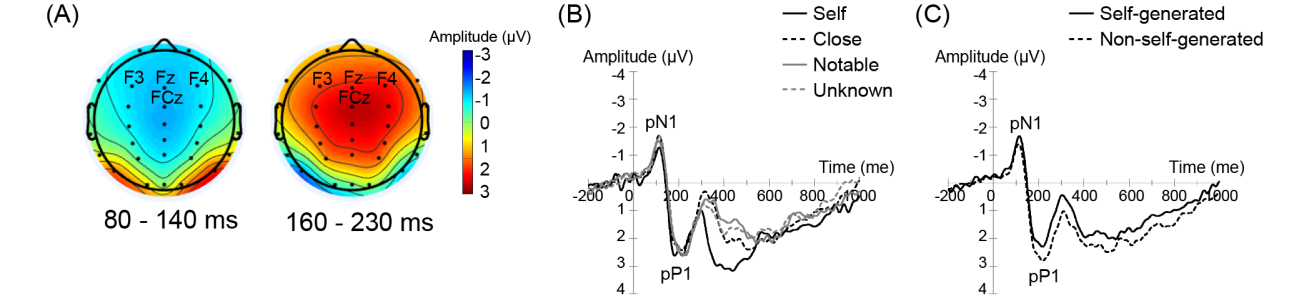
**

**Figure S2. pN1 and pP1 components.** (A) Topographical maps of pN1 (80–140 ms) and and pP1 (160–230ms) averaged across all experimental conditions. Grand-average waveforms for self- (black solid lines), close (black dashed lines), notable (gray solid lines), and unknown (gray dashed lines) name stimuli (B) and for self-generated (solid lines) and non-self-generated stimuli (C) at the frontal electrode sites (F3, Fz, F4, and FCz).
